# TreeQNet: a webserver for Treatment evaluation with Quantified Network

**DOI:** 10.1186/s12859-022-05024-y

**Published:** 2022-11-11

**Authors:** Zhenlei Li, Ya Huang, Qingrun Li, Yidi Sun, Chen Li, Jiarui Wu, Haoran Zheng, Rong Zeng

**Affiliations:** 1 School of Computer Science and Technology, University of Science and Technology of China, Jinzhai Road 96, Hefei, 230027 People’s Republic of China; 2 CAS Key Laboratory of Systems Biology, Shanghai Institute of Biochemistry and Cell Biology, Center for Excellence in Molecular Cell Science, Chinese Academy of Sciences, Shanghai, 200031 People’s Republic of China; 3 School of Life Science and Technology, ShanghaiTech University, Shanghai, 201210 People’s Republic of China; 4 Institute of Neuroscience, CAS Center for Excellence in Brain Science and Intelligence Technology, Chinese Academy of Sciences, Shanghai, 200031 People’s Republic of China; 5 Center for Single-Cell Omics, School of Public Health, Shanghai Jiao Tong University School of Medicine, Shanghai, 200025 People’s Republic of China; 6 College of Life Sciences, University of Chinese Academy of Sciences, Beijing, 100049 People’s Republic of China; 7 Anhui Key Laboratory of Software Engineering in Computing and Communication, University of Science and Technology of China, Jinzhai Road 96, Hefei, 230027 People’s Republic of China

**Keywords:** Quantified Network, Kinase-phospho substrate networks, Drug susceptibility prediction, Proteomic, Phosphoproteomic

## Abstract

**Background:**

Personalized therapy has been at the forefront of cancer care, making cancer treatment more effective. Since cancer patients respond individually to drug therapy, predicting the sensitivity of each patient to specific drugs is very helpful to apply therapeutic agents. Traditional methods focus on node (molecular) information but ignore relevant interactions among different nodes, which has very limited application in complex situations, such as cancer drug responses in real clinical practice.

**Results:**

Treatment evaluation with Quantified Network (TreeQNet) is a webserver which could predict sensitivity to drugs for patients through the innovative use of proteomic and phosphoproteomic network from tumor tissues.

**Conclusion:**

TreeQNet service: http://bioinfo.ustc.edu.cn/. TreeQNet source code: https://github.com/Really00/treeqnet-web-front/.

## Background

Cancer is one of the most common diseases threatening human health in the world. Drug treatment is most closely related to the specific origin of cancer and the location of gene mutation. Driven by the development of integrated *omics* technology, it becomes possible that select the most effective drug for each individual patient [1]. The main challenge of personalized medicine is to identify biomarkers for different cancers. Over the last decade, several methods have been developed to infer the potential relationships between cell line and drug [2]. Existing methods mainly rely on regression, classification or multi-kernel learning to predict drug response, but these methods are only based on single-molecule or static networks. Because the genetic backgrounds of within-class samples are heterogeneous and biochemical reactions are stochastic [3], single molecule biomarkers identified in differentially expressed molecules are limited in many cases. On the other hand, biological functions are often achieved through a set of collaborative molecules or a network of interacting molecules. Genes perform their functions by interacting with other molecules, and these interactions can be abstracted as edges of biomolecular networks. Therefore, it makes sense to introduce edge-based biomarkers [4]. Signaling pathways, protein complexes and sub-networks have greater discriminatory power than individual genes in distinguishing disease phenotypes [5]. In summary, a differentially expressed interaction or network can provide more details about human pathogenic states than a traditional single differentially expressed gene.

In a biological system, it is the interactions (regulations) or edges among molecules rather than single molecules that facilitate a biological function or signal transduction involved in diseases. Complex diseases are generally resulted from the failure of relevant systems instead of single molecules, which should be investigated in a dynamic and network manner. Thus, it is important to identify network signatures that are associated with complex diseases for early diagnosis and clinical prognosis. Edge biomarker represents one of the network signatures that are sensitive to network perturbation in diseases. Specifically, the “node” represents each single molecule, and the “edge” indicates the interaction or correlation between each pair of genes or molecules in the biological network. The “edge biomarker” is a more sensitive type of biomarker or signature that could reflect the perturbed networks in disease treatment. Cancer, as a complex disease caused by related systems, is more appropriate to use dynamic and intermolecular networks [6].

So if effective edge markers can be identified from the omics data may add new insights to cancer drug prediction based on the EdgeBiomarker algorithm proposed by Zhang et al. [4]. Combining the results of first-line drug trials in colorectal cancer using the miniPDX model [7], we innovatively use the proteomic and phosphoproteomic information of colorectal cancer patients to extract the kinase-substrate edges with the best classification ability as edge markers and construct a predictive model for patient sensitivity to three drugs, Afatinib, Gefitinib and Regorafenib [8]. Here, we develop TreeQNet, a web-based service for use by a broad range of researchers who hope to assist patients with their medication at the clinical level. TreeQNet service not only provides a new perspective for the study of colorectal cancer, but also for the study of other cancers.

## Methods and implementation

### Web server architecture

The TreeQNet web server is implemented using Model-View-View Model (MVVM) architecture model, which separates the user interface from the operation logic to provide users with a better experience. Javascript and Hypertext Markup Language (HTML) are the main languages for web services. The front-end web is based on HTML and Cascading Style Sheets (CSS). View layer is constructed based on Vue framework 2.5 which makes the page render and respond very quickly. Vue achieves efficient bidirectional data binding and flexible component system through MVVM idea, and provides a flexible file upload configuration. We develop Kexpress framework 1.0 based on express at the back-end.

**Fig. 1:**
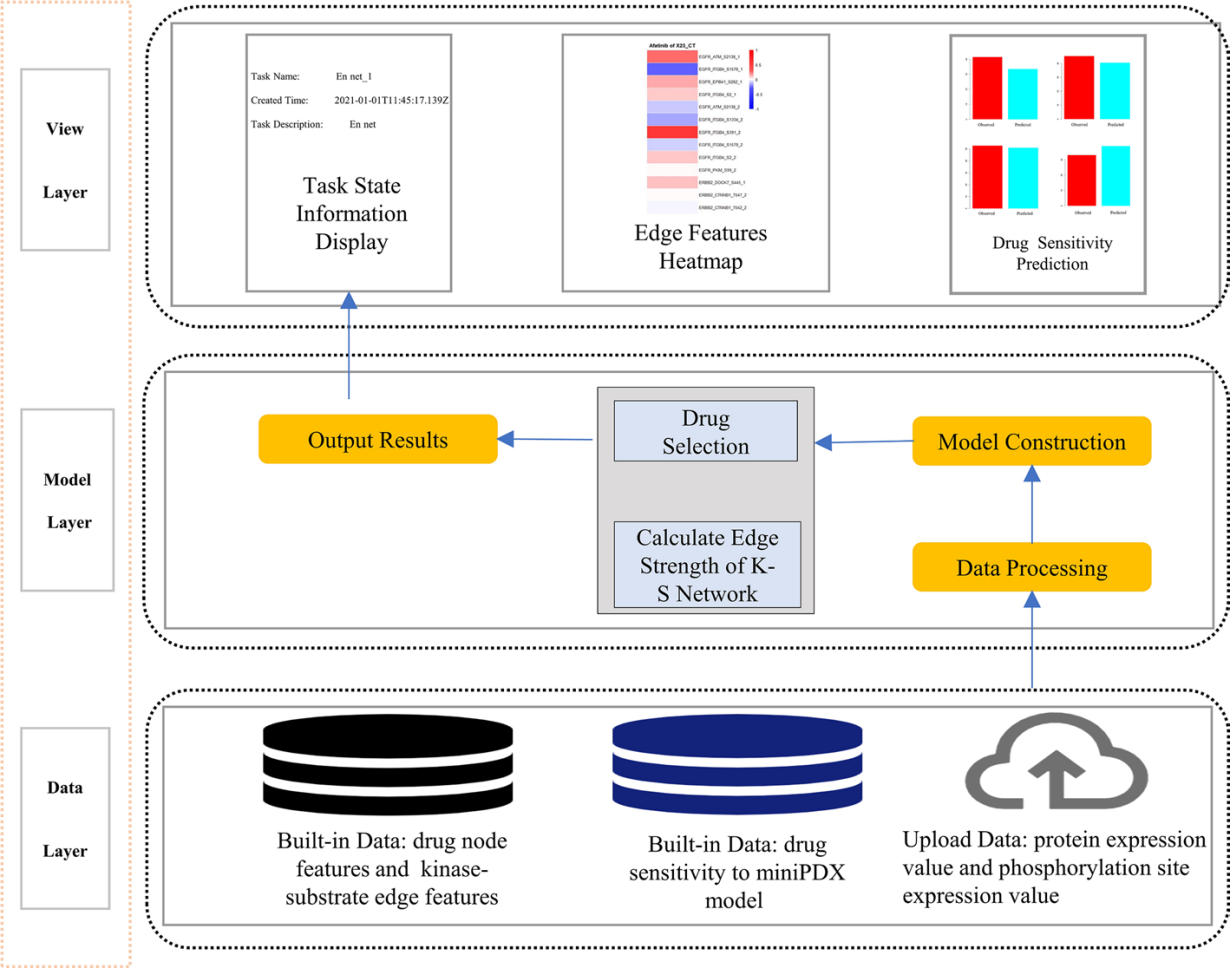
Web server architecture

Functionally, the server includes edge feature selection and drug sensitivity prediction. Model algorithm implemented with R seamlessly integrates with Kexpress framework shown in Fig. 1. The built-in data in the data layer includes the node data needed to predict the patients’ sensitivity to three drugs, edge data needed to predict sensitivity to three drugs, sensitivity results to three drugs in a miniPDX model from colorectal cancer patients, classification markers for colorectal cancer patients (1 indicates primary tumor, 2 indicates metastatic tumor). The upload data in the data layer is composed of protein expression data and phosphorylation site expression data uploaded by the users. In protein expression data file, the first column is the protein gene name of the patient to be predicted (no duplicate values) and the second column is the protein expression value of the patient to be predicted (after normalization, no missing values). This is similar for the phosphorylation site expression data file. Both files must be in csv format. Data processing mainly includes screening differentially correlated gene pairs (DCPs) by Pearson correlation coefficient (PCC) from 31 samples in the database as well as through the EdgeBiomarker algorithm for converting node data to edge data. In the process of EdgeBiomarker algorithm, the training set and testing set are divided by randomly sampling the dataset 100 times. Then the parameter alpha of the elastic network is searched in fixed steps by cross-validation method. Next, the parameter lambda corresponding to each alpha is then calculated and the best lambda is chosen for each alpha. Finally, the mean square error (MSE) is calculated and the features corresponding to the smallest MSE are then chosen as the edge biomarker. Model construction consists of converting all 31 samples in the database and the sample to be diagnosed into edge data, filtering edge features by the built-in kinase-substrate edge features in the system, further screening the edge features by correlation of the edge features and drug sensitivity as well as constructing an elastic network from these 31 processed samples. Ultimately, drug sensitivity prediction constructs a prediction model for the sensitivity of patients to three drugs, Afatinib, Regorafenib, and Gefitinib, which can realize the prediction of drug sensitivity in new patients.

### TreeQNet workflow

**Fig. 2:**
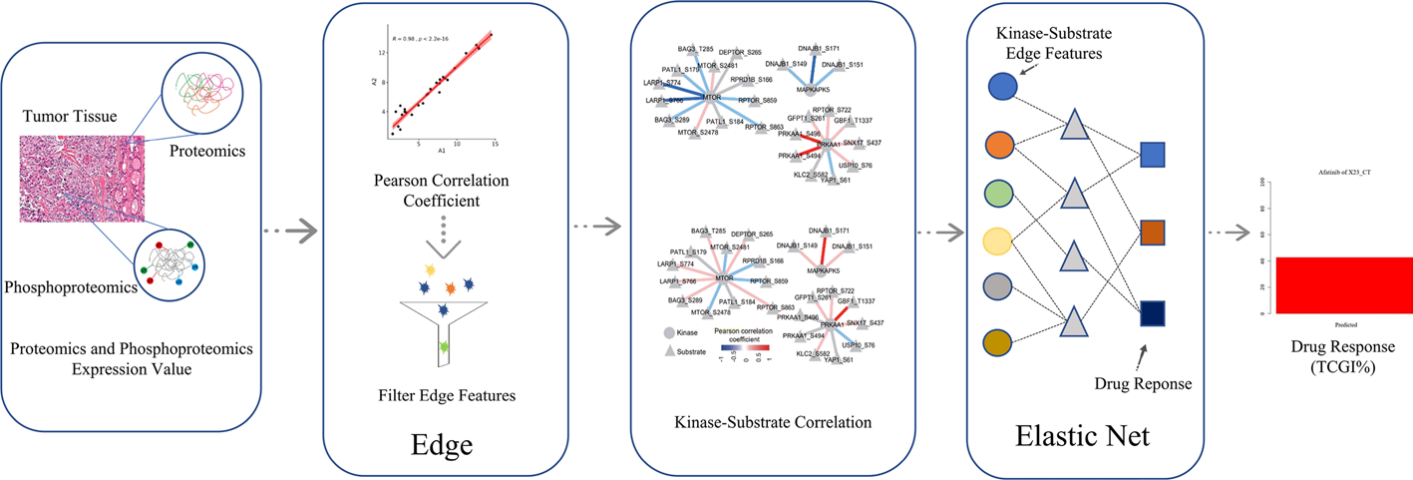
TreeQNet workflow

TreeQNet provides guidance on drug selection for individual patient, creating opportunities for personalized treatments of patients. TreeQNet can predict drug sensitivity based on kinase-substrate network and the whole algorithm flow is shown in Fig. 2. We first used EdgeBiomarker algorithm to construct kinase-substrate networks. After that, the edge strength between the kinase and the phosphorylated substrate is calculated based on PhosphoSitePlus [9] or NetworKIN 3.0 [10]. The edge transformations are made for the kinase and phosphorylated substrate data and the calculation method is shown in formula 1.

1 $$\begin{aligned} \begin{aligned} \text{ kinase, } u \text{ phospho } \text{- } \text{ substrate, } v\left( \begin{array}{l} x_{u, j, k} \\ x_{v, k, k} \end{array}\right) \quad -> \\ \text{ edge } \langle u-v\rangle _{k}\left( \frac{x_{u, j k}-\mu _{u, k}}{\sigma _{u, k}} \cdot \frac{x_{v, j, k}-\mu _{v, k}}{\sigma _{v, k}}\right) \end{aligned} \end{aligned}$$

where $x_{u, j, k}$ represents the expression value of the u-th kinase of the j-th sample in the k-th state. $x_{v, k, k}$ is the expression value of the vth substrate of the jth sample in the kth state, k is the state of the sample, set 1 as the primary tumor, and set 2 as the metastatic tumor. $\mu _{u, k}$ is the mean expression value of kinase u. $\mu _{v, k}$ is the mean expression value of substrate v. $\sigma _{u, k}$ is the standard deviation of the expression value of kinase u. $\sigma _{v, k}$ is the standard deviation of the expression value of substrate v.

These edge features are stored in the system as built-in data. Subsequently, we predict drug sensitivity based on elastic net regression model. Elastic net use 1696 edge strength features. Users can upload protein expression values and phosphorylation site expression values of patient tumor tissue and predict tumor cell growth inhibitions (TCGIs) for afatinib, gefitinib, and regorafenib.

### Example case

**Fig. 3:**
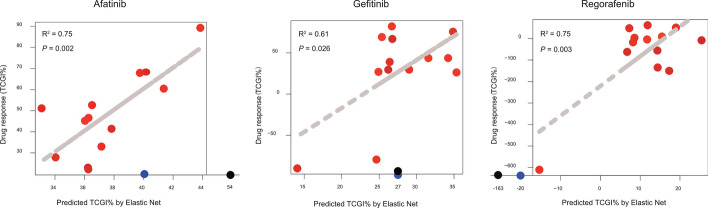
Correlation between predicted and observed TCGI %. The test set (red point) uses 13 CRC miniPDX models. Correlations and *p* values were calculated by Pearson’s method. Points on the coordinate axes are from the external colorectal cancer dataset, with blue points indicating a good prognosis and black points indicating a poor prognosis

To help users quickly get start with TreeQNet and show the usefulness of this tool, we provide 13 example cases for user reference. Users can download sample data in the database by “Download Example Data” button. The sample data contain protein expression value and phosphorylation site expression value data of 13 CRC patients. We measured the drug response effects of each tumor for each drug by tumor cell growth inhibition (TCGI). As shown in Fig. 3, the predicted results are obtained: Afatinib R-squared 0.75, *p* value 0.002, Gefitinib R-squared 0.61, *p* value 0.026 and Regorafenib R-squared 0.75, *p* value 0.003. The observed TCGI ratio varied a lot due to the limited number of samples. The prediction model to some extent could alleviate the influence of outliers and thus the predicted TCGIs showed narrower scales than the observed ones. While, the high correlation between predicted and observed ones suggested the good performance of our prediction model. In addition, two external colorectal cancer samples were used for testing (Fig. 3), other tumor and drug models will continue to be added and enhanced. Additional detailed user guides are available in the Additional file 1.

## Conclusion

TreeQNet is a user-friendly web server for treatment evaluation of cancer drugs that facilitates the precise treatment and evaluation for cancer patients and effectively selects the most suitable targeted therapy for patients without druggable mutations. For researchers, TreeQNet assists them to predict drug response through network analysis combined with proteomic and phosphoproteomic data. In addition, TreeQNet can help researchers establish an accurate index to determine the suitable drugs for a given tumor type. TreeQNet server accessible to users via web browsers is convenient for researchers to process data quickly, and provides a visual preview of the results.

## Supplementary Information

**Additional file 1. **Introduction to TreeQNet: The usage of the TreeQNet software is described. The format of the input data (including protein expression data and phosphorylation site expression data) is described. Relevant built-in data from the software is listed. We also outline the main calculation method and the results of typical run.

## Data Availability

TreeQNet web interface is freely available at http://bioinfo.ustc.edu.cn/. The source code can be found at https://github.com/Really00/treeqnet-web-front/.

## Abbreviations

TreeQNet: Treatment evaluation with Quantified Network
MVVM: Model-view-view model
HTML: Hypertext markup language
TCGIs: Tumor cell growth inhibitions
CRC: Colorectal cancer
