# Supplementary material for: TreeQNet: a webserver for Treatment evaluation with Quantified Network
Source: BMC Bioinformatics. 2022 Nov 11;23:473. doi: 10.1186/s12859-022-05024-y (PMC9652860; doi:10.1186/s12859-022-05024-y)
Supplement: Supplementary file 1 — Additional file 1. Introduction to TreeQNet: The usage of the TreeQNet software is described. The format of the input data (including protein expression data and phosphorylation site expression data) is described. Relevant built-in data from the software is listed. We also outline the main calculation method and the results of typical run. [file 12859_2022_5024_MOESM1_ESM.pdf]

Welcome to **TreeQnet**！

（Treatment Evaluation with Quantified Network）

Introduction to **TreeQnet**：

Cancer was one of the most dreaded diseases of the 20th century and continues to spread and increase its incidence in the 21st century, claiming millions of lives each year to the point that every quarter of people have a lifetime risk of developing cancer. While classic chemotherapeutics directly target the DNA of cells, contemporary anticancer drugs involve molecularly targeted therapy, such as targeting proteins that are abnormally expressed in cancer cells. Targeted therapy has been successful in some malignancies, but its effectiveness is often limited by drug resistance and side effects on normal tissues and cells, and none is 100% effective against same cancer in all people. Cancer patients have variable responses to drug therapy, and predicting patient sensitivity to a particular drug in advance can help medical institutions and patients better choose therapeutic drugs.

At present, most of the research on this in the world focuses on screening differentially expressed molecules from tens of thousands of molecular omics characteristics of patients, combining machine learning and existing data to train and select the data, and obtain a set of molecules with the best classification effect as Markers, and

then carry out cell or animal model validation, so as to diagnose and infer the drug sensitivity of patients. However, traditional thinking not only ignores non-differentially expressed molecules but also can only use node (molecular) information, resulting in a great waste of high-throughput data and limited application to complex situations such as tumor drug sensitivity in real clinical patients. This requires us to use a new perspective to discover effective biomarkers and build models.

Based on the point-edge transformation algorithm proposed by Zhang in 2014 (Zhang et al., 2014), we innovatively use the proteome and phosphoproteomics information of colorectal cancer patients to construct edge expression values, then elastic network was used to extract the kinase-substrate edge with the best classification ability as the edge marker and build a prediction model for the sensitivity of patients to Afatinib, Regorafenib, and Gefitinib (Li et al., 2020). The prediction effect on our dataset is shown in Fig.1. For unknown samples even if it is a single sample, we only need to use edge transformation to convert the point data of the sample into edge data, and then use our existing prediction model to predict the drug sensitivity of patients.

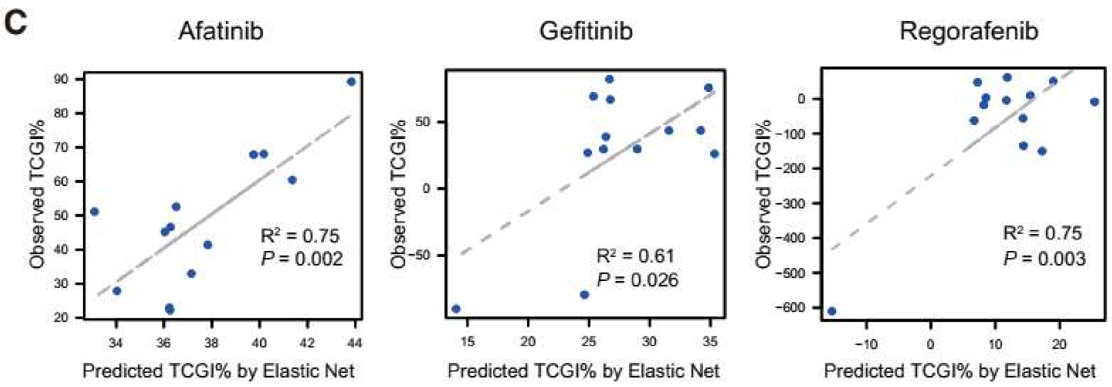

Fig.1: In a validation set of 13 CRC miniPDX models based on kinase-phosphosubstrate edge features, correlation between predicted and observed TCGI %. Correlations and p-values were calculated by Pearson's method.

Here, we develop TreeQNet, a web-based service for use by a broad range of researchers who hope to assist patients with their medication at the clinical level. In the future, we will develop more high-throughput omics and network models to predict more clinical states.

Reference:

1. Zhang W, Zeng T, Chen L. EdgeMarker: Identifying differentially correlated molecule pairs as edge-biomarkers. J Theor Biol. 2014 Dec 7;362:35-43.

2. Li C, Sun YD, Yu GY et al. Integrated Omics of Metastatic Colorectal Cancer. Cancer Cell. 2020 Nov 9;38(5):734-747.

Here are all related files:

Input (strictly follow the specified format of the documentation):

Step 1: Select the drug you want to predict, currently only support the prediction of three drugs: Afatinib, Regorafenib, and Gefitinib.

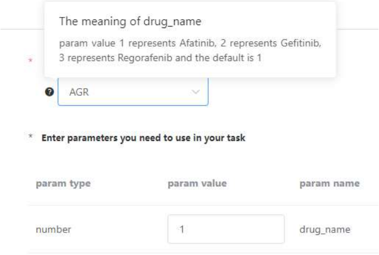

Step 2: Upload protein and phosphorylation site files.

pro.csv： the protein expression value data file of the patient tumor tissue (any tumor can be) to be predicted. The first column is the protein gene name of the patient to be predicted (no duplicate values). The second column is the protein expression value of the patient to be predicted (after normalization, no missing values). The column names are [ID] and [patient ID (numbers or letters can be used freely, and must be the same as the number in pho.csv)]. The uploaded file must be in csv format.

	A	B	C
1	ID	23_CT	
2	ND2	0.197117	
3	ND1	-0.2421	
4	ND5	0.446455	
5	ND6	1.266235	
6	ND4	0.870645	
7	COX3	0.773424	
8	ND3	-0.40152	
9	ATP8	1.246935	
10	FLJ10292	0.933519	
11	RBM8	0.978338	
12	RHAG	-0.70711	

pho.csv： the data file of phosphorylation site expression value of the tumor tissue of the patient to be predicted (any tumor can be. The first column is the ID of the phosphorylation site of the patient to be predicted (there cannot be duplicate values), the format is [gene

name]_[phosphorylation site category][phosphorylation site position]. The second column is the expression value of the phosphorylation site of the patient to be predicted (after normalization, no missing value), the column names are [ID] and [patient ID (numbers or letters can be used freely, and must be the same as the one in pro.csv) same number)]. The uploaded file must be in csv format.

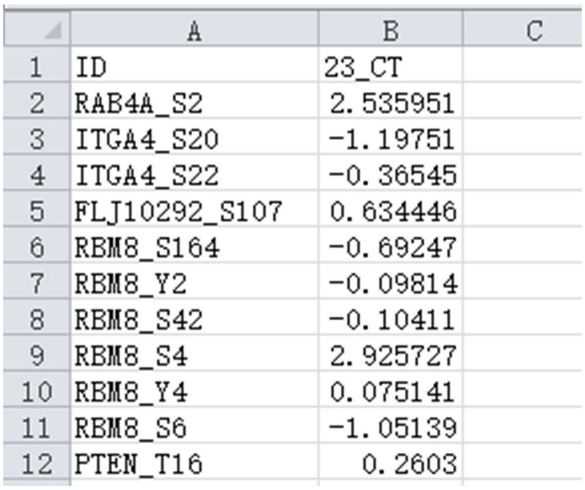

	A	B	C
1	ID	23_CT	
2	RAB4A_S2	2.535951	
3	ITGA4_S20	-1.19751	
4	ITGA4_S22	-0.36545	
5	FLJ10292_S107	0.634446	
6	RBM8_S164	-0.69247	
7	RBM8_Y2	-0.09814	
8	RBM8_S42	-0.10411	
9	RBM8_S4	2.925727	
10	RBM8_Y4	0.075141	
11	RBM8_S6	-1.05139	
12	PTEN_T16	0.2603	

Built-in data：

node_afatinib.csv: Node data needed to predict afatinib sensitivity.

node_gefitinib.csv: Node data needed to predict gefitinib sensitivity.

node_regorafenib.csv: Node data needed to predict regorafenib sensitivity.

edata_afatinib.csv: Edge data needed to predict sensitivity to afatinib.

edata_gefitinib.csv: Edge data needed to predict sensitivity to gefitinib

edata_regorafenib.csv: Edge data needed to predict sensitivity to regorafenib.

drug_afatinib.csv: Sensitivity results to afatinib in a miniPDX model in

colorectal cancer patients.

drug_gefitinib.csv: Sensitivity results to gefitinib in a miniPDX model in colorectal cancer patients.

drug_regorafenib.csv: Sensitivity results to regorafenib in a miniPDX model in colorectal cancer patients.

labl.csv: Classification markers for colorectal cancer patients, 1 indicates primary tumor，2 indicates metastatic tumor.

Version 1.0 *can only support the prediction of the three drugs* ***Afatinib****,* ***Regorafenib*** *and* ***Gefitinib*** *based on protein and phosphorylated protein!*

calculation method：

The following edge transformations are made for the kinase and phosphorylated substrate data:

$$\begin{matrix} kinase,\ u \\ phospho-substrate,\ v \end{matrix} \begin{pmatrix} x_{u,j,k} \\ x_{v,j,k} \end{pmatrix} \quad -> \ edge\langle u-v\rangle_k \left(\frac{x_{u,j,k}-\mu_{u,k}}{\sigma_{u,k}} \cdot \frac{x_{v,j,k}-\mu_{v,k}}{\sigma_{v,k}} \right)$$

$x_{u,j,k}$ represents the expression value of the u-th kinase of the j-th sample in the k-th state,

$x_{v,j,k}$ represents the expression value of the vth substrate of the jth sample in the kth state, k represents the state of the sample, set 1 as the primary tumor, and set 2 as the metastatic tumor.

$$\mu_{u,k} = \frac{1}{n_k}\sum_{j=1}^{n_k} x_{u,j,k}$$ is the mean expression value of kinase u.

$$\mu_{v,k} = \frac{1}{n_k}\sum_{j=1}^{n_k} x_{v,j,k}$$ is the mean expression value of substrate v.

$$\sigma_{u,k} = \sqrt{\frac{1}{n_k}\sum_{j=1}^{n_k}\left(x_{u,j,k} - \mu_{u,k}\right)^2}$$ is the standard deviation of the expression value of kinase u.

$$\sigma_{v,k} = \sqrt{\frac{1}{n_k}\sum_{j=1}^{n_k}\left(x_{v,j,k} - \mu_{v,k}\right)^2}$$ is the standard deviation of the expression value of substrate v.

Then, the edge data is screened and trained by the elastic network model, and the edge features established by the selected kinases and phosphorus substrates are obtained, and then the drug sensitivity prediction of unknown samples is carried out.

Results:

1. The drug sensitivity prediction map of the patient to be predicted.

Image captions and colors automatically change based on user-entered drug type and sample name and sensitivity. If

sensitivity > 0, output as red image (Figure 2), If sensitivity < 0, output as blue image (Figure 3).

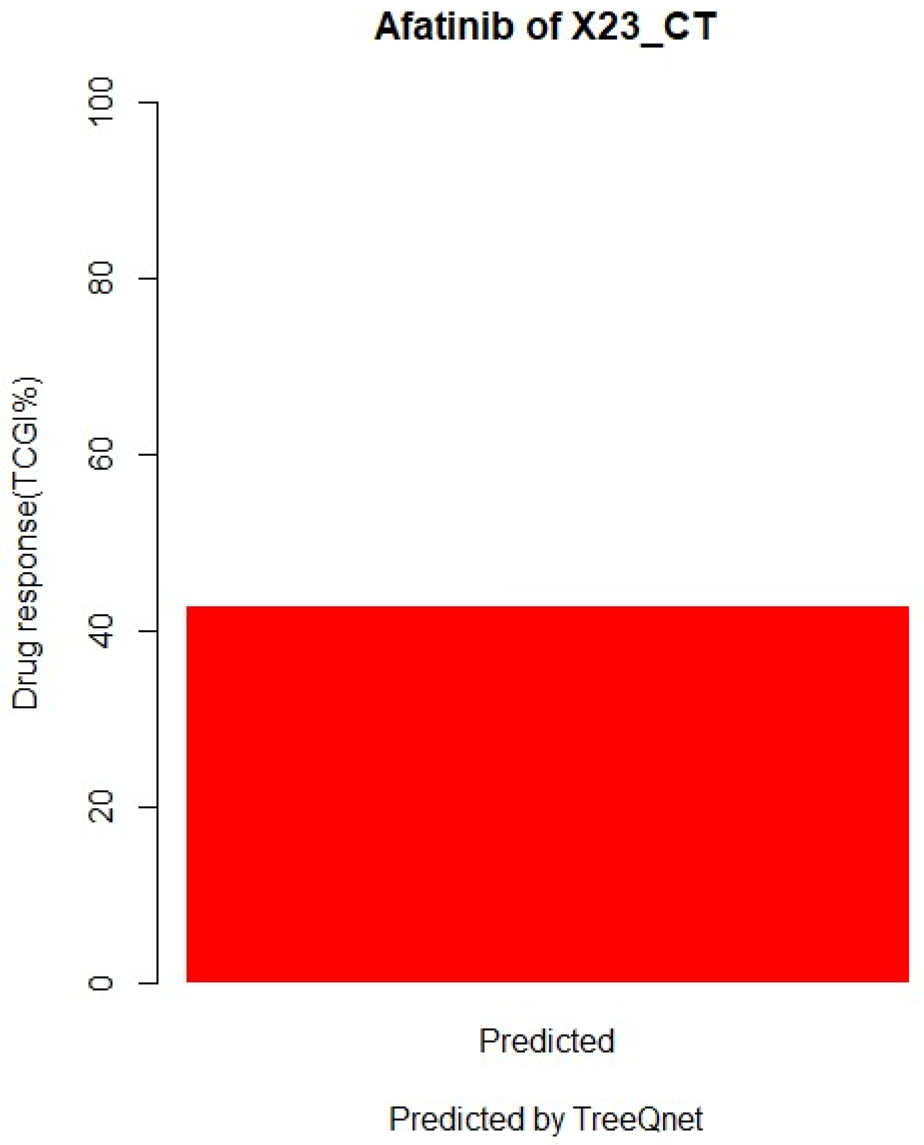

Figure 2. Sensitivity to afatinib in patients to be predicted > 0

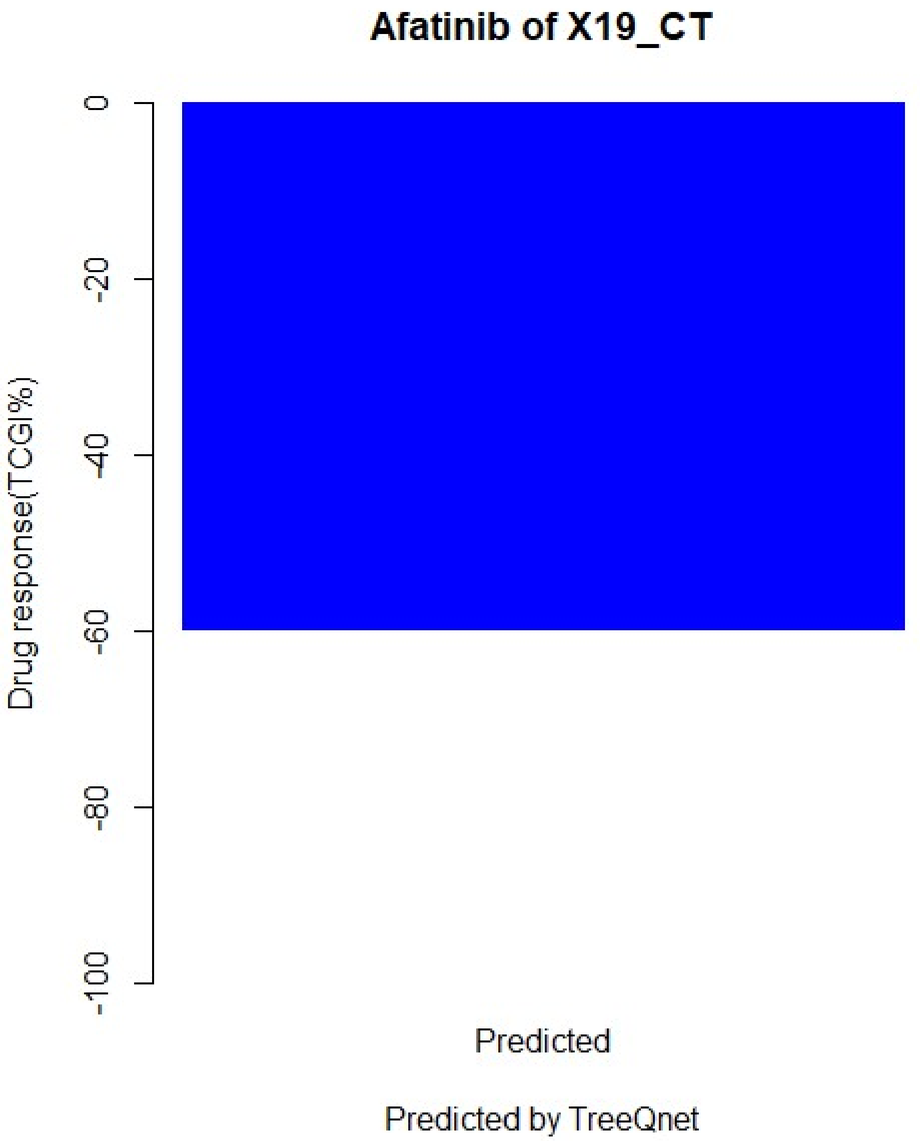

Figure 3. Sensitivity to afatinib in patients to be predicted < 0

2. Heatmap of edge features after training (Figure 4)

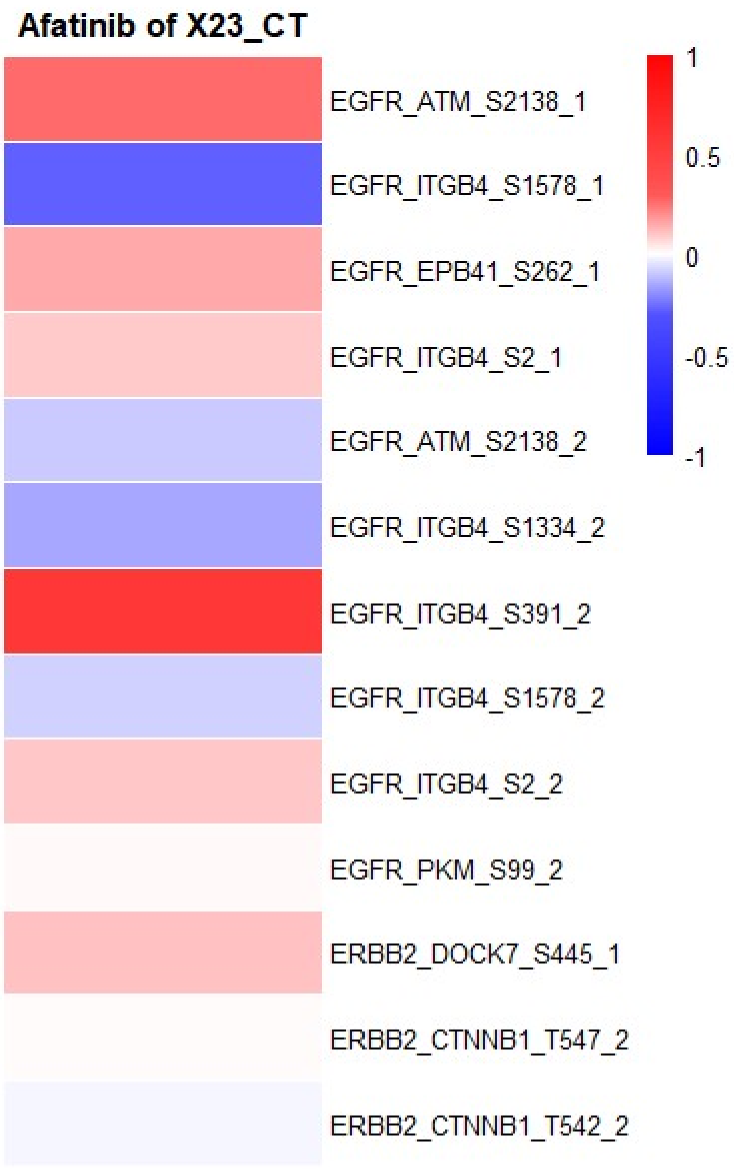

Figure 4. Eigenvalues between selected kinase-phosphate substrate edge features and drug response to afatinib in patients to be predicted.
